# *N*-Phenyl­adamantane-1-sulfinamide

**DOI:** 10.1107/S1600536808019570

**Published:** 2008-07-05

**Authors:** Mrityunjoy Datta, Alan J Buglass, Chang Seop Hong, Jeon Hak Lim

**Affiliations:** a Department of Chemistry, Korea Advanced Institute of Science and Technology, Daejeon 305-701, Republic of Korea; b Department of Chemistry, Korea University, Seoul 136-701, Republic of Korea

## Abstract

In the racemic title compound, C_16_H_21_NOS, the mol­ecules are packed into polymeric chains in the *b-*axis direction and are linked along the *b* axis by N—H⋯O and C—H⋯O hydrogen bonds.

## Related literature

For literature on *N*-alkyl­alkanesulfinamides, see: Sato *et al.* (1975 ▶), Schuckmann *et al.* (1978 ▶); Ferreira *et al.* (2005 ▶). For related literature on cyclic *N*-aryl­alkanesulfinamides (sultims), see: Schulze *et al.* (2005 ▶). For the synthesis, see: Stretter *et al.* (1969 ▶). For related literature, see: Han *et al.* (2002 ▶); Weix & Ellman (2003 ▶).
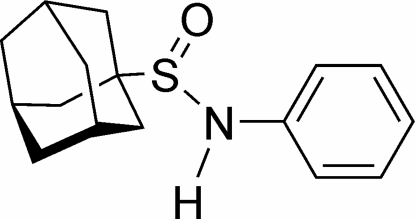


## Experimental

### 

#### Crystal data

C_16_H_21_NOS
*M* *_r_* = 275.40
Monoclinic, 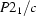

*a* = 11.6614 (2) Å
*b* = 14.5582 (3) Å
*c* = 9.0632 (2) Å
β = 109.7770 (10)°
*V* = 1447.90 (5) Å^3^
*Z* = 4
Mo *K*α radiation
μ = 0.22 mm^−1^
*T* = 293 (2) K
0.12 × 0.08 × 0.06 mm

#### Data collection

Bruker APEXII diffractometer
Absorption correction: multi-scan (*SADABS*; Sheldrick, 1996 ▶) *T*_min_ = 0.975, *T*_max_ = 0.987
14147 measured reflections
3563 independent reflections
2623 reflections with *I* > 2σ(*I*)
*R*_int_ = 0.029

#### Refinement

*R*[*F* ^2^ > 2σ(*F* ^2^)] = 0.041
*wR*(*F* ^2^) = 0.126
*S* = 1.07
3563 reflections
172 parameters
H-atom parameters constrained
Δρ_max_ = 0.28 e Å^−3^
Δρ_min_ = −0.30 e Å^−3^

### 

Data collection: *APEX2* (Bruker, 2001 ▶); cell refinement: *SAINT* (Bruker, 2001 ▶); data reduction: *SAINT*; program(s) used to solve structure: *SHELXTL* (Sheldrick, 2008 ▶); program(s) used to refine structure: *SHELXL97* (Sheldrick, 2008 ▶); molecular graphics: *ORTEP-3* (Farrugia, 1997 ▶) and *DIAMOND* (Brandenburg, 1998 ▶); software used to prepare material for publication: *WinGX* (Farrugia, 1999 ▶).

## Supplementary Material

Crystal structure: contains datablocks I, global. DOI: 10.1107/S1600536808019570/lx2059sup1.cif

Structure factors: contains datablocks I. DOI: 10.1107/S1600536808019570/lx2059Isup2.hkl

Additional supplementary materials:  crystallographic information; 3D view; checkCIF report

## Figures and Tables

**Table 1:** Hydrogen-bond geometry (Å, °)

*D*—H⋯*A*	*D*—H	H⋯*A*	*D*⋯*A*	*D*—H⋯*A*
N1—H1⋯O1^i^	0.86	2.17	2.988 (2)	160
C10—H10*A*⋯O1^i^	0.97	2.35	3.305 (2)	168

Symmetry code: (i) 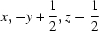.

**Fig. 1.**
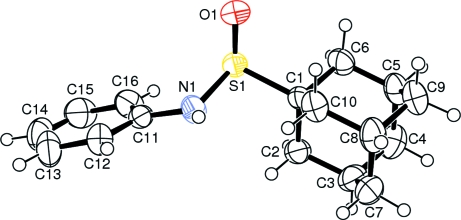
Molecular structure of (I), with atom labels and 50% probability displacement ellipsoids for non-H atoms.

**Fig. 2.**
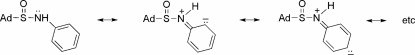
Resonance structures for (I).

**Fig. 3.**
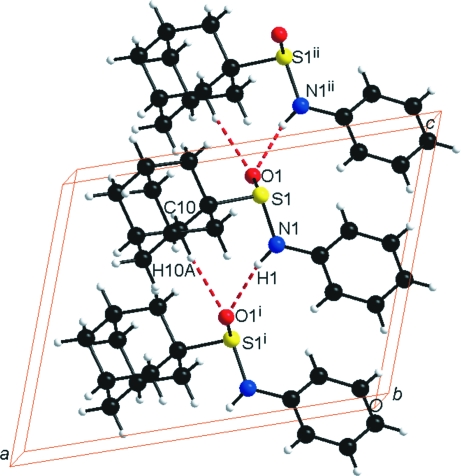
The packing of (I), viewed down the b axis, showing hydrogen bonding [symmetry code: (i) x, -y + 1/2, z - 1/2; (ii) x, -y + 1/2, z + 1/2].

## supplementary crystallographic information

### Comment

The title compound (I) was prepared from aniline and 1-adamantanesulfinyl chloride, which was itself prepared from adamantane and thionyl chloride in the presence of anhydrous AlCl_3_ (Stretter *et al.*, 1969).

The molecular structure of (I) (Fig. 1) resembles those of *N*-alkylsulfinamides, except that the *N*-(aryl)C bond (1.409 Å) is considerably shorter than typical *N*-(alkyl)C bonds in *N*-alkylsulfinamides (1.470–1.530 Å) (Sato *et al.*, 1975; Schuckmann *et al.*, 1978; Ferreira *et al.*, 2005). The short bond suggests significant delocalization of electrons over the nitrogen atom and the benzene ring. This can be interpreted as indicating considerable contributions to the overall structure of (I) from resonance structures such as those in Fig. 2. The molecules of (I) (with alternating (*R*) and (*S*) configurations) are packed in a chain along the *b* axis (Fig. 3). The crystal packing (Fig. 3) is stabilized by intermolecular N—H···O and C—H···O hydrogen bonds (Fig. 3 and Table 1; symmetry code as in Fig. 3). Interest in sulfinamides lies mainly in their performance as chiral building blocks in organic synthesis (Han *et al.*, 2002; Weix and Ellman, 2003).

### Experimental

Compound (I) was prepared by the method of Stretter *et al.*(1969), using aniline (424 mg, 4.56 mmol), 1-adamantanesulfinyl chloride (500 mg, 2.28 mmol) and anhydrous diethyl ether (30 ml). Column chromatography (silica gel, ethyl acetate-dichloromethane, 1:9) gave (I) as white crystals (610 mg, 97%) mp 427–428 K. Literature mp was 428 K (Stretter *et al.*, 1969). Single crystals suitable for X-ray analysis were obtained by evaporation of a solution of the title compound (I) in dichloromethane at room temperature. Spectroscopic analysis: FTIR (KBr) (cm^-1^) 3179, 2908, 2851, 1595, 1488, 1450, 1285, 1228, 1175, 1063, 1034, 877. ^1^H NMR (400 MHz, CDCl_3_, p.p.m. with respect to TMS) 7.26–7.22 (m, 2H), 7.01–6.97 (m, 3H), 5.43 (bs, 1H), 2.18 (s, 3H), 1.92 (dd, J = 11.8, 22.8 Hz, 6H), 1.74 (dd, J = 12.2, 23.4 Hz, 6H). ^13^C NMR (100 MHz, CDCl_3_, p.p.m. with respect to TMS) 142.4, 129.2, 122.4, 117.9, 58.2, 36.3, 34.6, 28.5. EIMS m/*z* (%) 276 (MH^+^,39), 275 (*M*^+^, 85), 259(*M*^+^–16, 16), 228 (18), 227 (*M*^+^–SO,75), 136 (59), 135 (*M*^+^–PhNHSO, 100), 107 (28), 93 (MH^+^–adamantanesulfinyl, 66), 79 (61).

### Refinement

H atoms were located on a difference Fourier map geometrically and refined using a riding model with N—H = 0.86 Å, C—H = 0.93–0.98 Å and with *U*_iso_(H) = 1.2 times *U*_eq_(C, N).

### Crystal data

**Table:**

C_16_H_21_NOS	*F*_000_ = 592
*M**_r_* = 275.40	*D*_x_ = 1.263 Mg m^−^^3^
Monoclinic, *P*2_1_/*c*	Mo *K*α radiation λ = 0.71073 Å
Hall symbol: -P 2ybc	Cell parameters from 4542 reflections
*a* = 11.6614 (2) Å	θ = 2.3–18.3º
*b* = 14.5582 (3) Å	µ = 0.22 mm^−^^1^
*c* = 9.0632 (2) Å	*T* = 293 (2) K
β = 109.7770 (10)º	Block, white
*V* = 1447.90 (5) Å^3^	0.12 × 0.08 × 0.06 mm
*Z* = 4	

### Data collection

**Table:**

Bruker APEXII diffractometer	3563 independent reflections
Radiation source: fine-focus sealed tube	2623 reflections with *I* > 2σ(*I*)
Monochromator: graphite	*R*_int_ = 0.030
*T* = 293(2) K	θ_max_ = 28.3º
ω scans	θ_min_ = 2.3º
Absorption correction: multi-scan(SADABS; Sheldrick, 1996)	*h* = −15→15
*T*_min_ = 0.975, *T*_max_ = 0.987	*k* = −18→19
14147 measured reflections	*l* = −12→12

### Refinement

**Table:**

Refinement on *F*^2^	Secondary atom site location: difference Fourier map
Least-squares matrix: full	Hydrogen site location: inferred from neighbouring sites
*R*[*F*^2^ > 2σ(*F*^2^)] = 0.041	H-atom parameters constrained
*wR*(*F*^2^) = 0.126	*w* = 1/[σ^2^(*F*_o_^2^) + (0.0675*P*)^2^ + 0.1762*P*] where *P* = (*F*_o_^2^ + 2*F*_c_^2^)/3
*S* = 1.07	(Δ/σ)_max_ = 0.001
3563 reflections	Δρ_max_ = 0.28 e Å^−^^3^
172 parameters	Δρ_min_ = −0.30 e Å^−^^3^
Primary atom site location: structure-invariant direct methods	Extinction correction: none

### Special details

**Table:**

Geometry. All e.s.d.'s (except the e.s.d. in the dihedral angle between two l.s. planes) are estimated using the full covariance matrix. The cell e.s.d.'s are taken into account individually in the estimation of e.s.d.'s in distances, angles and torsion angles; correlations between e.s.d.'s in cell parameters are only used when they are defined by crystal symmetry. An approximate (isotropic) treatment of cell e.s.d.'s is used for estimating e.s.d.'s involving l.s. planes.
Refinement. Refinement of *F*^2^ against ALL reflections. The weighted *R*-factor *wR* and goodness of fit *S* are based on *F*^2^, conventional *R*-factors *R* are based on *F*, with *F* set to zero for negative *F*^2^. The threshold expression of *F*^2^ > σ(*F*^2^) is used only for calculating *R*-factors(gt) *etc*. and is not relevant to the choice of reflections for refinement. *R*-factors based on *F*^2^ are statistically about twice as large as those based on *F*, and *R*- factors based on ALL data will be even larger.

### Fractional atomic coordinates and isotropic or equivalent isotropic displacement parameters (Å^2^)

**Table:**

	*x*	*y*	*z*	*U*_iso_*/*U*_eq_	
C1	0.58983 (13)	0.39058 (9)	0.82150 (16)	0.0364 (3)	
C2	0.56242 (15)	0.48043 (11)	0.7296 (2)	0.0484 (4)	
H2A	0.5187	0.5217	0.7757	0.058*	
H2B	0.5117	0.4685	0.6219	0.058*	
C3	0.68250 (16)	0.52473 (12)	0.7334 (2)	0.0580 (5)	
H3	0.6652	0.5824	0.6741	0.070*	
C4	0.76125 (18)	0.54460 (12)	0.9024 (3)	0.0638 (5)	
H4A	0.8368	0.5736	0.9047	0.077*	
H4B	0.7188	0.5863	0.9496	0.077*	
C5	0.78874 (16)	0.45500 (12)	0.9943 (2)	0.0535 (4)	
H5	0.8397	0.4677	1.1029	0.064*	
C6	0.66905 (15)	0.41030 (12)	0.99233 (18)	0.0481 (4)	
H6A	0.6261	0.4511	1.0403	0.058*	
H6B	0.6861	0.3535	1.0517	0.058*	
C7	0.74864 (17)	0.45997 (14)	0.6590 (2)	0.0619 (5)	
H7A	0.8239	0.4881	0.6583	0.074*	
H7B	0.6983	0.4476	0.5514	0.074*	
C8	0.77682 (15)	0.37019 (13)	0.7515 (2)	0.0536 (4)	
H8	0.8203	0.3288	0.7034	0.064*	
C9	0.85636 (16)	0.38968 (13)	0.9200 (2)	0.0579 (4)	
H9A	0.9326	0.4173	0.9220	0.069*	
H9B	0.8748	0.3327	0.9789	0.069*	
C10	0.65764 (15)	0.32443 (11)	0.7496 (2)	0.0473 (4)	
H10A	0.6077	0.3099	0.6427	0.057*	
H10B	0.6754	0.2678	0.8094	0.057*	
S1	0.44633 (4)	0.34441 (3)	0.82885 (4)	0.04623 (15)	
O1	0.47547 (12)	0.25339 (9)	0.90826 (15)	0.0702 (4)	
N1	0.37398 (12)	0.32958 (9)	0.63941 (15)	0.0463 (3)	
H1	0.4153	0.3178	0.5790	0.056*	
C11	0.24605 (13)	0.33580 (9)	0.57455 (18)	0.0390 (3)	
C12	0.18648 (15)	0.29240 (11)	0.4345 (2)	0.0499 (4)	
H12	0.2305	0.2573	0.3866	0.060*	
C13	0.06152 (17)	0.30068 (14)	0.3647 (2)	0.0657 (5)	
H13	0.0223	0.2726	0.2687	0.079*	
C14	−0.00505 (17)	0.35041 (14)	0.4370 (3)	0.0686 (6)	
H14	−0.0893	0.3546	0.3917	0.082*	
C15	0.05395 (17)	0.39325 (14)	0.5752 (3)	0.0654 (5)	
H15	0.0094	0.4275	0.6235	0.078*	
C16	0.17819 (16)	0.38689 (13)	0.6449 (2)	0.0542 (4)	
H16	0.2169	0.4169	0.7393	0.065*	

### Atomic displacement parameters (Å^2^)

**Table:**

	*U*^11^	*U*^22^	*U*^33^	*U*^12^	*U*^13^	*U*^23^
C1	0.0339 (7)	0.0456 (7)	0.0304 (7)	0.0010 (6)	0.0117 (6)	0.0018 (6)
C2	0.0400 (8)	0.0553 (9)	0.0519 (9)	0.0083 (7)	0.0180 (7)	0.0142 (7)
C3	0.0517 (10)	0.0570 (10)	0.0698 (12)	0.0027 (8)	0.0264 (9)	0.0205 (9)
C4	0.0553 (11)	0.0540 (10)	0.0831 (14)	−0.0106 (8)	0.0248 (10)	−0.0099 (9)
C5	0.0447 (9)	0.0632 (10)	0.0455 (9)	−0.0046 (7)	0.0060 (8)	−0.0122 (8)
C6	0.0499 (10)	0.0593 (9)	0.0335 (8)	−0.0017 (7)	0.0121 (7)	−0.0036 (7)
C7	0.0453 (10)	0.0912 (13)	0.0554 (10)	−0.0031 (9)	0.0252 (9)	0.0080 (9)
C8	0.0359 (9)	0.0692 (10)	0.0574 (10)	0.0059 (7)	0.0180 (8)	−0.0127 (8)
C9	0.0376 (9)	0.0674 (11)	0.0607 (11)	0.0053 (8)	0.0061 (8)	−0.0033 (9)
C10	0.0412 (9)	0.0542 (9)	0.0446 (8)	0.0038 (7)	0.0121 (7)	−0.0094 (7)
S1	0.0400 (2)	0.0676 (3)	0.0334 (2)	−0.00510 (17)	0.01537 (18)	0.00352 (16)
O1	0.0590 (8)	0.0870 (9)	0.0588 (8)	−0.0153 (7)	0.0123 (7)	0.0304 (7)
N1	0.0355 (7)	0.0683 (9)	0.0359 (7)	0.0016 (6)	0.0133 (6)	−0.0037 (6)
C11	0.0358 (8)	0.0410 (7)	0.0413 (8)	0.0004 (6)	0.0142 (7)	0.0068 (6)
C12	0.0423 (9)	0.0498 (9)	0.0552 (10)	−0.0023 (7)	0.0131 (8)	−0.0067 (7)
C13	0.0449 (10)	0.0674 (11)	0.0719 (13)	−0.0087 (9)	0.0031 (9)	−0.0068 (10)
C14	0.0361 (10)	0.0729 (12)	0.0899 (16)	0.0032 (8)	0.0122 (11)	0.0168 (11)
C15	0.0509 (11)	0.0761 (12)	0.0751 (14)	0.0205 (9)	0.0292 (10)	0.0158 (10)
C16	0.0498 (10)	0.0646 (10)	0.0500 (10)	0.0118 (8)	0.0192 (8)	0.0012 (8)

### Geometric parameters (Å, °)

**Table:**

C1—C10	1.525 (2)	C8—C10	1.536 (2)
C1—C2	1.526 (2)	C8—H8	0.980
C1—C6	1.538 (2)	C9—H9A	0.970
C1—S1	1.825 (2)	C9—H9B	0.970
C2—C3	1.531 (2)	C10—H10A	0.970
C2—H2A	0.970	C10—H10B	0.970
C2—H2B	0.970	S1—O1	1.491 (1)
C3—C7	1.513 (3)	S1—N1	1.651 (1)
C3—C4	1.523 (3)	N1—C11	1.409 (2)
C3—H3	0.980	N1—H1	0.860
C4—C5	1.522 (3)	C11—C12	1.377 (2)
C4—H4A	0.970	C11—C16	1.388 (2)
C4—H4B	0.970	C12—C13	1.384 (2)
C5—C9	1.530 (2)	C12—H12	0.930
C5—C6	1.535 (2)	C13—C14	1.378 (3)
C5—H5	0.980	C13—H13	0.930
C6—H6A	0.970	C14—C15	1.360 (3)
C6—H6B	0.970	C14—H14	0.930
C7—C8	1.527 (3)	C15—C16	1.374 (3)
C7—H7A	0.970	C15—H15	0.930
C7—H7B	0.970	C16—H16	0.930
C8—C9	1.521 (2)		
			
C10—C1—C2	110.6 (1)	C9—C8—C7	109.7 (2)
C10—C1—C6	109.0 (1)	C9—C8—C10	109.5 (1)
C2—C1—C6	109.5 (1)	C7—C8—C10	109.8 (2)
C10—C1—S1	113.4 (1)	C9—C8—H8	109.3
C2—C1—S1	108.1 (1)	C7—C8—H8	109.3
C6—C1—S1	106.1 (1)	C10—C8—H8	109.3
C1—C2—C3	109.1 (1)	C8—C9—C5	109.2 (1)
C1—C2—H2A	109.9	C8—C9—H9A	109.9
C3—C2—H2A	109.9	C5—C9—H9A	109.9
C1—C2—H2B	109.9	C8—C9—H9B	109.9
C3—C2—H2B	109.9	C5—C9—H9B	109.9
H2A—C2—H2B	108.3	H9A—C9—H9B	108.3
C7—C3—C4	110.0 (2)	C1—C10—C8	108.6 (1)
C7—C3—C2	109.1 (2)	C1—C10—H10A	110.0
C4—C3—C2	109.8 (1)	C8—C10—H10A	110.0
C7—C3—H3	109.3	C1—C10—H10B	110.0
C4—C3—H3	109.3	C8—C10—H10B	110.0
C2—C3—H3	109.3	H10A—C10—H10B	108.3
C5—C4—C3	109.4 (1)	O1—S1—N1	109.79 (8)
C5—C4—H4A	109.8	O1—S1—C1	106.43 (7)
C3—C4—H4A	109.8	N1—S1—C1	99.34 (6)
C5—C4—H4B	109.8	C11—N1—S1	121.5 (1)
C3—C4—H4B	109.8	C11—N1—H1	119.3
H4A—C4—H4B	108.3	S1—N1—H1	119.3
C4—C5—C9	109.6 (2)	C12—C11—C16	118.7 (2)
C4—C5—C6	109.6 (1)	C12—C11—N1	119.2 (1)
C9—C5—C6	109.5 (1)	C16—C11—N1	122.0 (1)
C4—C5—H5	109.4	C11—C12—C13	120.4 (2)
C9—C5—H5	109.4	C11—C12—H12	119.8
C6—C5—H5	109.4	C13—C12—H12	119.8
C5—C6—C1	109.0 (1)	C14—C13—C12	120.3 (2)
C5—C6—H6A	109.9	C14—C13—H13	119.9
C1—C6—H6A	109.9	C12—C13—H13	119.9
C5—C6—H6B	109.9	C15—C14—C13	119.2 (2)
C1—C6—H6B	109.9	C15—C14—H14	120.4
H6A—C6—H6B	108.3	C13—C14—H14	120.4
C3—C7—C8	109.6 (1)	C14—C15—C16	121.3 (2)
C3—C7—H7A	109.7	C14—C15—H15	119.4
C8—C7—H7A	109.7	C16—C15—H15	119.4
C3—C7—H7B	109.7	C15—C16—C11	120.1 (2)
C8—C7—H7B	109.7	C15—C16—H16	120.0
H7A—C7—H7B	108.2	C11—C16—H16	120.0

### Hydrogen-bond geometry (Å, °)

**Table:**

*D*—H···*A*	*D*—H	H···*A*	*D*···*A*	*D*—H···*A*
N1—H1···O1^i^	0.86	2.17	2.988 (2)	160
C10—H10A···O1^i^	0.97	2.35	3.305 (2)	168

Symmetry codes: (i) *x*, −*y*+1/2, *z*−1/2.
